# Biological functions of CDK5 and potential CDK5 targeted clinical treatments

**DOI:** 10.18632/oncotarget.14538

**Published:** 2017-01-06

**Authors:** Alison Shupp, Mathew C. Casimiro, Richard G. Pestell

**Affiliations:** ^1^ Departments of Cancer Biology, Medical Oncology, Sidney Kimmel Cancer Center, Thomas Jefferson University, Philadelphia, PA, USA; ^2^ Pennsylvania Cancer and Regenerative Medicine Research Center, Baruch S Blumberg Institute, Doylestown, PA, USA

**Keywords:** CDK5, cancer

## Abstract

Cyclin dependent kinases are proline-directed serine/threonine protein kinases that are traditionally activated upon association with a regulatory subunit. For most CDKs, activation by a cyclin occurs through association and phosphorylation of the CDK’s T-loop. CDK5 is unusual because it is not typically activated upon binding with a cyclin and does not require T-loop phosphorylation for activation, even though it has high amino acid sequence homology with other CDKs. While it was previously thought that CDK5 only interacted with p35 or p39 and their cleaved counterparts, Recent evidence suggests that CDK5 can interact with certain cylins, amongst other proteins, which modulate CDK5 activity levels. This review discusses recent findings of molecular interactions that regulate CDK5 activity and CDK5 associated pathways that are implicated in various diseases. Also covered herein is the growing body of evidence for CDK5 in contributing to the onset and progression of tumorigenesis.

## INTRODUCTION

Cyclin dependent kinases are proline-directed serine/threonine protein kinases that are traditionally activated upon association with a regulatory subunit. CDKs are a part of a kinase family that has been conserved throughout evolution and can be found in species from *Saccharomyces cerevisia* to humans. In humans there are 13 different CDKs (CDK1 - CDK13) that are highly expressed in mitotic cells [1]. For most CDKs, activation by a cyclin occurs through association and phosphorylation of the CDK's T-loop. Despite having high amino acid sequence homology with other CDKs, CDK5 is unusual because it is not typically activated upon binding with a cyclin and does not require T-loop phosphorylation for activation. Additionally, CDK5 has functions in both terminally differentiated and proliferating cells [2]. CDK5 was first identified in 1992 by multiple groups and was given a different name by each, including tau kinase II [3], neuronal Cdc2 like kinase [4], brain proline-directed kinase [5], PSSALRE [6], and CDK5 [7]. An isoform of CDK5, termed either CDK5-SV or CDK5-V1, was recently discovered [8, 9]. One study reported that this splice variant lacks 32 amino acids encoded by exon 7 [8], while another study stated the missing 32 amino acids are encoded by exon 6 [9]. Although these two groups reported conflicting data, it has been suggested that the identified isoforms are in fact the same protein and the variances in their data are due to different methodologies [10].

CDK5 can be mapped to chromosome 7q36 and its expression is upregulated by the transcription factors Fos and CREB through the MEK/ERK pathway and by δFosB [11, 12]. CDK5 plays a vital role in the central nervous system but has functions in other cell types. Outside of the nervous system, active CDK5 has been found in pancreatic β cells [13], corneal epithelial cells [14] and monocytes [15] amongst various other cell types [10, 16]. In the nervous system, CDK5 is involved in neuron migration, neurite outgrowth and support, and synaptogenesis. CDK5's function in cells other than neurons includes the induction of cell motility, apoptosis, and cell cycle progression as well as functions involved with the immune system, lymphatic system, vascularization, and insulin secretion. A summary of CDK5 functions as discussed herein can be found in Table I. CDK5 has recently been implicated in diseases, including the development and progression of cancer and neurodegenerative diseases. For this reason, the regulation of CDK5 activity is now emerging as a candidate therapeutic target.

**Table 1:** Cyclin dependent kinase 5's functions in various biological systems and cellular processes

Biological system/process	CDK5 function	Mechanism
Central nervous system	Support growth cones	CDK5 phosphorylates CRMP2A at Ser27 during semaphorin3A stimulation. CDK5 also phosphorylates neurofilament heavy chain to promote neurofilament assembly [33–35]
	Growth cone collapse	CDK5 associates with alpha2-chimerin and phosphorylates CRMP2 at Ser522. CRMP2 further phosphorylated and inactivated by GSK3beta [37]
Immune system	Increased IFNγ-induced PD-L1 expression	CDK5 expression decreases the expression of PD-L1 transcriptional repressors (IRF2 and IRF2BP) [55]
Insulin secretion	Reduction of insulin secretion	CDK5 phosphorylates L-VDCC and prevents exocytosis of insulin [13]
Vascular	Promotes angiogenesis	CDK5 expression increases abundance of HIF-1α [53]
Lymphatic	Lymphatic valve formation	CDK5 phosphorylates Foxc2, which regulates the expression of connexin 37 [59]
Cell Cycle	Increased expression of cyclins and other CDK's	Rb is a downstream target of CDK5's activity [50]
	Reduction of CDK5 activity	Cyclin D1 and cyclin E can bind CDK5 to prevent CDK5's activation [39, 40]
Cancer Progression	Cell proliferation	Reduction of p25 expression or CDK5 expression can prevent proliferation [50]
	Cell migration/metastasis	CDK5 activity leads to caldesmon phosphorylation and actin polymerization. CDK5 enhances pro-migratory P13K/AKT signaling [61, 62]

## CDK5 ACTIVATORS AND REGULATORS

Unlike other CDKs, CDK5 is not primarily activated by cyclins. Instead it is through specific binding with the proteins p35 or p39, or their respective cleaved counterparts p25 and p29, that CDK5 becomes active [1, 17, 18]. It was found that *p35* knockout mice have defective cortical lamination and adults suffered from sporadic lethality and seizures [19], which is a less severe phenotype than that exhibited by *Cdk5* knockout mice [20]. *p39*-/- mice did not display any obvious abnormalities, however *p35/p39* compound knockout mice displayed a phenotype identical to that of the *Cdk5*-/- mice [21], suggesting that while p39 may not play a pivotal role in Cdk5 activation, it becomes necessary for nervous system development in the absence of p35.

p35 has a myristolation sequence that localizes it to phospholipid membranes [22]. Active CDK5 can phosphorylate p35 at Ser8 and Thr138. In the brain, phosphorylation of S8 is constant throughout development, but phosphorylation of T138 is found more abundantly in fetal brain tissue [23]. The phosphorylation at S8 leads to a more diffuse localization throughout the cytoplasm. This could be due to increased p35 mobility on membranes due to an altered interaction between the protein and phospholipids that constitute cell membranes [24]. p35 phosphorylation at T138 prevents its cleavage to p25 by calpain [23]. Because CDK5 has various regulatory functions in neuron development and migration, it is likely that the phosphorylation of p35 at T138 protects against aberrant CDK5 activation through formation of p25 in the fetal stage of brain development when CDK5 activity is also high [1]. Additionally, *in vitro*, under conditions of oxidative stress, p35 has been found conjugated to SUMO2 at Lys246 and Lys290, which led to increased p35/CDK5 activity [25].

As previously mentioned, the CDK5 activator p25 is formed through cleavage of p35 by calpain. This produces both the p25 product as well as a p10 product. Cleavage of p35 occurs under stress conditions such as amyloidβ presence, excitotoxicity, or oxidative stress [22, 26]. This cleavage allows p25 to localize to nuclear and perinuclear regions by removing the p10 myristolation sequence [22]. Compared with p35, p25 has a longer half-life, and therefore prolongs the activation period of CDK5, leading to increased phosphorylation of CDK5's target proteins [22, 27].

The functions of CDK5 activators p39 and p29 largely overlap with those of p35 and p25, respectively, however their expression throughout brain regions vary. p39 and p29 are mainly expressed in postnatal cerebral cortex and the hindbrain while p35 and p25 are largely expressed in the cerebral cortex of developing brains [27]. The localization of p39 to membranes is similar to that of p35 due to its conserved myristolation sequence [22]. Likewise, p39 also shows a more diffuse localization upon phosphorylation of Ser8 by CDK5 [24]. p39 can be phosphorylated by CDK5 at Ser173, a site equivalent to T138 in p35, and Thr84, however the effect of these phosphorylations on controlling protein stability have not yet been explored [1, 24].

In addition to p35 and p39, cyclin I has also been shown to activate CDK5. Cyclin I-CDK5 binding targets CDK5 to the nucleus [28] and increases levels of anti-apoptotic proteins Bcl2 and Bcl2l1 via the MEK/ERK pathway [29]*.* This upregulation of Bcl2 and Bcl211 is observed only through cyclin I activation of CDK5, not activation via p35 [29, 30]. CDK5 has been found to bind cyclin D1 and cyclin D3 in human fibroblasts, however this interaction had no influence on the activation and kinase activity of CDK5 [7, 31].

**Figure 1:**
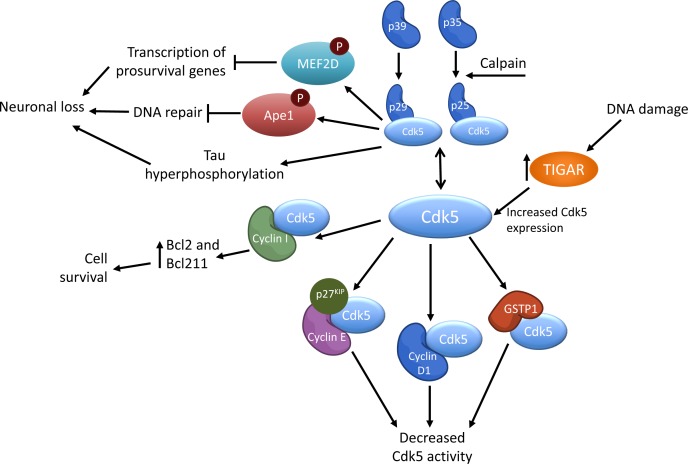
Simplified schematic of the regulation of CDK5 activity

While CDK5 is only activated by p35/p25, p39/p29, or cyclin I, the activity of CDK5 can be modulated by a variety of other proteins, as depicted in Figure 1. For instance, cyclin D1 can attenuate CDK5 kinase activity by competing with p35 for binding with CDK5, thereby forming an inactive complex of cyclin D1 and CDK5 (Fig. 1). CDK5 and cyclin D1 can be found in the rat cerebellum during the first 24 days of postnatal development, albeit at varying abundances. CDK5 abundance increased while cyclin D1 decreased from day 9 on to adulthood [32]. In post-mitotic neurons, cyclin D1/CDK5 association was found to lead to cell cycle related neuronal apoptosis through sustained MEK/ERK signaling [33].

Cyclin E can directly interact with Cdk5 to reduce its activity. Cyclin E was found to sequester mouse Cdk5 away from other protein activators along with p27^KIP1^. The formation of this complex, and consequent attenuation of Cdk5 activity was found to promote synaptic plasticity, memory formation, and dendritic growth, as *cyclin E*-/- mice, that had increased Cdk5 activity, were deficient in these processes [34]. While this result may seem counterintuitive due to active CDK5's function with supporting neurite outgrowths, this observation could be explained by an overabundance of active CDK5 detrimentally effecting neurite outgrowth and subsequently synaptic plasticity. This theory would be consistent with findings that CDK5 expression levels are increased in certain neurodegenerative diseases, and that it is the aberrant CDK5 activity that leads to neurite collapse and death [35–37].

Glutathione-S-transferase (GSTP1) is another regulator of CDK5 activity that functions by competing with p35 for CDK5 binding. GSTP1 also reduces aberrant CDK5 activity by scavenging for molecules associated with oxidative stress and thereby decreasing the likelihood of p35/p39 cleavage to p25/p29 [38] (Figure 1).

TP53 induced glycolysis regulatory phosphatase (TIGAR) has been shown to upregulate *CDK5* expression levels in the presence of induced DNA damage (Figure 1). Knockdown of *TIGAR* led to decreased *CDK5* expression, decreased phosphorylated ATM, and consequently increased levels of induced DNA damage. This suggests that DNA damage repair is mediated via TIGAR activation of the CDK5-ATM pathway [39].

## CDK5 IN CELL CYCLE AND OTHER PATHWAYS

Previously, CDK5 was thought to function in a cell cycle independent manner; however, recently the retinoblastoma protein (Rb) was discovered as a downstream target of CDK5. Expression of CDK5 leads to the phosphorylation of Rb, ultimately leading to the expression of cyclins and other cdks [40]. The protein kinase CK1 is phosphorylated by CDK5, and is involved in a wide array of signaling pathways including cell cycle, DNA repair, and apoptosis [41]. When CDK5 phosphorylates CK1, its kinase activity is subsequently reduced [42]. The functional affect of CDK5-mediated phosphorylation of CK1 on cell cycle, DNA repair, or apoptosis has yet to be explored.

**Figure 2:**
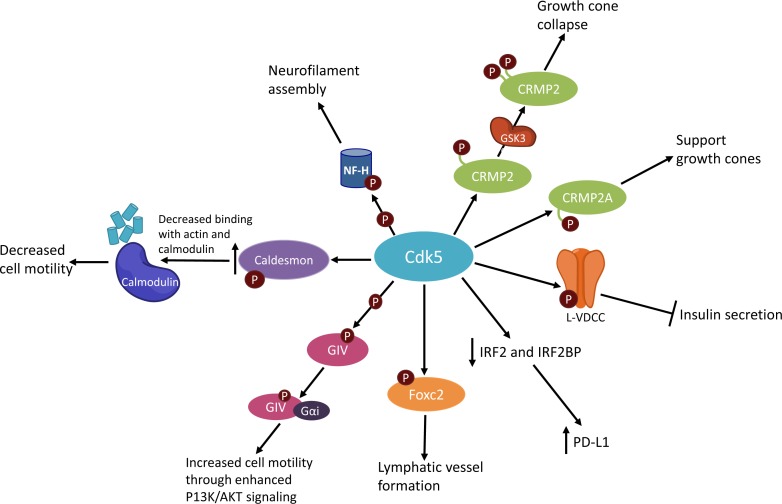
Simplified schematic of CDK5 activities

In pancreatic β cells, CDK5 activity reduces insulin secretion in response to glucose abundance (Figure 2). This was demonstrated using CDK5 inhibitors, as well as inhibition of CDK5's activator p35. When CDK5 is active, it phosphorylates the L-type voltage-dependent Ca^+2^ channel (L-VDCC) at Ser783, which prevents the association of L-VDCC with syntaxin and SNAP-25, thereby preventing exocytosis of insulin from the cell [13].

Within the immune system, CDK5 has been implicated in IFNγ-induced programmed death ligand 1 (PD-L1) upregulation, which allows certain cells to evade detection by the immune system. Decreased CDK5 expression led to increased expression of the PD-L1 transcriptional repressors IRF2 and IRF2BP and consequent decreased PD-L1 expression (Figure 2) [43]. PD-L1 is a ligand that binds with PD-1, which is found on various immune cells. The binding of PD-L1 and PD-1 decreases an immune response by inhibiting T-cell activation and cytokine production. In normal tissues this is vital for maintaining homeostasis [44]. However tumor cells can also express PD-L1, which allows them to avoid detection and elimination by T-cells [45, 46].

CDK5 promotes the formation of lymphatic vessels. CDK5 phosphorylates Foxc2, a protein that regulates the expression of connexin 37, which is critical for lymphatic valve formation (Figure 2). Moreover, knockout of *CDK5* in the endothelium leads to lymphedema formation and embryonic lethality in mice [47].

CDK5 has previously been implicated in the migration of neurons. *CDK5* knockout mice have abnormal cortical lamination, and more than 60% of *CDK5*-/- mice died *in utero* [20]. Various studies have since implicated CDK5 in cell migration as it governs cancer metastasis. In prostate cancer cells, inhibition of CDK5 by the drug roscovitine prevented cell migration. The roscovitine treated cells did not project lamellopodia, and had reduced tubulin structures compared to untreated cells. This suggests that CDK5 inhibition prevented the establishment of cell polarity required for movement [48]. Additionally, knockdown of *CDK5* in melanoma cell lines decreased cell motility and cell spreading *in vitro*, and decreased formation of lung and liver metastases *in vivo* in a mouse model of human melanoma. The decrease in *CDK5* expression led to decreased phosphorylation of caldesmon, which decreased its binding affinity with actin and calmodulin (Figure 2) [49]. Another mechanism by which CDK5 may promote cell migration is by enhancing pro-migratory P13K/AKT signaling. CDK5 phosphorylates the Gα –interacting vesicle associated protein (GIV), which promotes GIV interaction with Gαi, thereby enhancing P13K/AKT signaling (Figure 2) [50]. Together, these studies demonstrate the importance of CDK5 in cell motility, a naturally occurring and necessary process. However, CDK5 mediated movement could also be an underlying driver of cancer metastasis and could be targeted in treatments to halt cancer metastasis.

## CYTOSKELETAL ORGANIZATION

An important function of CDK5, especially in neurons, is the organization of the cytoskeleton and support of cellular outgrowths (Figure 2). Expression of p35 or p39 *in vitro* stimulates neurite outgrowths, and a dominant negative mutant of CDK5 was found to abolish the formation of these outgrowths [51]. CDK5 supports axon and neurite outgrowth is through phosphorylation of the neurofilament heavy chain, resulting in the assembly of neurofilaments [52].

CDK5 has been shown to both prevent and promote growth cone collapse under different circumstances. CDK5 phosphorylates the protein CRMP2A at Ser27, which can be stabilized by Pin1 to support the growth of growth cones in the presence of semaphorin3A stimulation [53, 54]. Additionally, CDK5 can promote axonal growth through indirect activation of CRMP2 by phosphorylating the protein Axin. Phosphorylated Axin inhibits GSK3β activity, leading to an increase in active, unphosphorylated CRMP2 [55] (Figure 2).

Conversely, CDK5 promotes the collapse of growth cones through association with CRMP2 and α2-chimerin, an adaptor protein between CRMP2 and CDK5-p35. This association of CRMP2, α2-chimerin, and CDK5-p35 promotes the phosphorylation of CRMP2 at Ser522 by CDK5. In turn, this allows for CRMP2 to associate with and be phosphorylated at T514 by GSK3β, resulting in CRMP2 inactivation, microtubule disassembly, and ultimately growth cone collapse [56]. In this manner, CDK5 activity can both prevent and promote collapse of growth cones.

CDK5 can also reduce cellular outgrowth by regulating cytoskeletal organization through phosphorylation of p35 at T138, which prevents the polymerization of microtubules. This phosphorylation at T138 is found primarily in fetal brain tissues as opposed to adult brain [23].

## ROLE OF CDK5 IN NEUROLOGICAL DISEASE

Due to the many roles of CDK5 in the development of the nervous system, as well as the effects of cellular stress on CDK5 activation, CDK5 has been implicated in the progression of various neurological diseases and as a potential therapeutic target in disease treatment. For instance, while CDK5 normally phosphorylates collapsin response mediator protein 2 (CRMP2) to stimulate axon growth, it was found that hyperphosphorylation of CRMP2, as well as Tau, were implicated in the generation of neurofibrillary tangles characteristic of Alzheimer's disease [53]. Cell stress, including the presence of amyloid beta, is known to aberrantly activate CDK5 due to the formation of p25, which has been shown to cause the hyperphosphorylation of Tau, leading to atypical cell cycling, synaptotoxicity, and neuronal apoptosis [57]. Additionally, increased CDK5 activity caused by the sumoylation of p35 under oxidative stress, also contributes to neurodegeneration [25].

While CDK5 overexpression and aberrant activation are associated with neurodegenerative diseases, a loss or reduction in CDK5 activity is implicated in certain intellectual disabilities and neurodevelopmental disorders. Decreased CDK5 activity has been associated with intellectual disability in NF1 microdeletion syndrome patients [58] and schizophrenia [59]. Additionally, transgenic mice with decreased Cdk5 activity exhibited spontaneous seizures [60] as well as behaviors similar to ADHD [61].

## CDK5 EXPRESSION IN CANCER

Elevated levels of CDK5 have been found in various mouse tumors and human malignant tumors [40] [53, 62–65]. The mechanisms involve effects on angiogenesis, cell proliferation and the immune system. As noted above, CDK5 enhances pRb phosphorylation and thereby cell-cycle progression [40]. Furthermore, CK1 is phosphorylated by CDK5, which in turn governs cell cycle, DNA repair, and apoptosis [41]. Increased levels of CDK5 target proteins are being considered as possible biomarkers of specific cancers. For example, an increase in CRMP2 phosphorylation could be a potential biomarker for certain lung cancers, as phosphorylated CRMP2 was found in the nuclei of biopsied lung cancer cells, but not cells in the surrounding epithelium [53].

In a transgenic mouse model of sporadic medullary thyroid carcinoma (MTC), p25 overexpression led to the development of bilateral malignant thyroid tumors, and was fatal after 30 weeks. However, arresting p25 expression at 5, 11, or 16 weeks led to 100 percent survival in all mice analyzed after 30 weeks. Similar results were discovered *in vitro*, in which reducing p25 expression or knocking down Cdk5 expression prevented further cell proliferation. This suggests that it is the aberrant activation of Cdk5 by p25 that leads to the progression of sporadic MTC [40].

CDK5 expression in medulloblastoma allows tumor cells to evade detection by T-cells *in vivo*. Conversely, decreased CDK5 expression enhanced the recruitment of CD4^+^ T-cells to the tumor site in mice, and increased the tumor-free survival rate of the mice. CDK5 regulates the evasion of tumors from the immune system by decreasing expression of transcriptional repressors of PD-L1 expression, thus increasing the abundance of PD-L1 [45].

Inhibiting CDK5 activity in hepatocellular carcinoma (HCC) cells prevented angiogenesis *in vivo* by decreasing the abundance of HIF-1α. Because HCC is a highly vascularized tumor type, inhibiting CDK5 and therefore angiogenesis, could prove a promising treatment for this tumor subtype and other highly vascularized tumors [64].

## CDK5 AS A TARGET FOR DISEASE TREATMENT

Due to the biological and clinically relevant importance of CDK5's function in multiple cell types, CDK5 presents an attractive therapeutic target for treating a variety of conditions such as diabetes, cancer, and neurodegeneration. Additionally, the upregulation of *CDK5* associated with various cancers and neurodegenerative diseases further implicates its role in the development and progression of disease. Recently, tamoxifen (TMX), a drug currently used in breast cancer treatment, was found to decrease CDK5 activation by competitively binding with p35 and p25, and preventing their activation of CDK5. While the TMX inhibition of CDK5 activity could contribute to the anti-tumor effects of the drug, TMX treatment was also found to decrease Tau phosphorylation, suggesting a use for tamoxifen in treating Alzheimer's disease [66]. However, because of the broad functions of CDK5 in different cell and tissue types and the pan CDK inhibitory effect on other family members, the off target affects of a CDK5 inhibitory drug may create undesirable side effects. Nonetheless, CDK inhibitors are an intriguing clinical therapy for the treatment of various cancers. A list of current cyclin-dependent kinase inhibitors, including inhibitors of CDK5, and their associated clinical trials for the treatment of cancer can be seen in Table II.

**Table II:** Previous and ongoing cancer clinical trials using cyclin dependent kinase inhibitors [64, 65]

Treatment	Major Targets	Disease(s)	Clinical trial identifier
Terameprocol	CDK1	Phase I: Leukemia, refractory solid tumors, lymphoma, glioma	NCT00664677, NCT00664586, NCT00404248
PHA-793887	CDK1, CDK2, CDK4	Phase I: Solid tumors	NCT00996255
Flavopiridol	CDK1, CDK2, CDK4, CDK7, CDK9	Phase I-II: Various cancer including leukemia, multiple myeloma, lymphoma, sarcoma, and solid tumors (alone and in combination with other cytotoxic drugs)	NCT02520011, NCT00112723, NCT00005974, NCT00098579, NCT00007917, NCT00324480
BAY1000394	CDK1, CDK2, CDK4, CDK9	Phase I: solid tumors	NCT01188252
Dinaciclib	CDK1, CDK2, CDK5, CDK9	Phase I-II: Advanced malignancies and relapsed multiple myeloma (alone and in combination with other cytotoxic drugs)	NCT01783171, NCT01624441, NCT01096342, NCT02684617, NCT01434316, NCT00871663, NCT01624441
P276-00	CDK1, CDK4, CDK9	Phase I-II: Multiple myeloma, mantle cell lymphoma, head and neck cancers, cyclin D1-positive melanoma	NCT00882063, NCT00848050, NCT00824343, NCT00899054, NCT00835419
AT7519	CDK2, CDK4, CDK5, CDK9	Phase I: Advanced or metastatic solid tumors, lymphoma	NCT02503709, NCT01652144, NCT01627054
R-roscovitine	CDK2, CDK5	Phase I-II: Advanced solid tumors, non-small cell lung cancer	NCT00999401, NCT00372073
SNS-032	CDK2, CDK7, CDK9	Phase I: B-lymphoid malignancies and advanced solid tumors	NCT00446342
P1446A-05	CDK4	Phase I: Advanced refractory solid tumors and hematological tumors	NCT00840190 NCT00772876
PD 0332991	CDK4, CDK6	Phase I: Advanced cancers, mantle cell lymphoma Phase II: Multiple myeloma, advanced breast cancer, non-small cell lung cancer, ovarian cancer	NCT01522989, NCT00141297, NCT02008734, NCT02101034, NCT01976169, NCT01907607, NCT01356628, NCT01291017, NCT01536743
LY2835219	CDK4, CDK6	Phase I-II: Metastatic breast cancer, non small cell lung cancer	NCT02102490, NCT02246621, NCT02441946, NCT02450539, NCT02079636, NCT02779751 NCT02152631, NCT02675231

This is representative rather than a comprehensive list of past and present clinical trials in the field.

One of the most well studied CDK inhibitors being used in cancer clinical trials is flavopiridol, a drug developed by Tolero pharmaceuticals under the name Alvocidib. Flavopiridol was found to competivively bind to the ATP-binding pocket of CDK1, CDK2, CDK4, and CDK9, consequently inducing apoptosis in both dividing and quiescent cells. Early clinical trials with flavopiridol as a monotherapy proved ineffective in that there was a narrow window between no clinical response and severe, lethal tumor lysis. Ongoing trials involve combination therapies with other novel chemotherapy agents to overcome the limitations of flavorpiridol [67].

Another relatively well studied CDK inhibitor, Dinaciclib, was found to be more efficacious than flavopiridol, with IC_50_ values in the low nanomolar range (1-4 nM – in various models flavopiridol's IC50 values range from 50-350 nM) [67, 68]. Dinaciclib selectively inhibits CDK1, CDK2, CDK5, and CDK9 [67]. Preclinical studies and early clinical trials demonstrated the cytotoxicity of Dinaciclib in solid tumors and chronic lymphocytic leukemia, while not affecting T-cell function or number [69].

Roscovitine, marketed under the name Seliciclib, is an inhibitor of CDK5 and CDK2. Many of the clinical trials for Seliciclib were intiated determine dose-limiting toxicities of the drug alone or in combination with other chemotherapeutics. While roscovitine is used widely experimentally to inhibit CDK5 activity, it is not being intensively examined as a clinical cancer therapeutic [67].

To potentially reduce broad undesirable off target effects of pan-CDK inhibitors, CDK5 inhibitory peptide (CIP) has been studied as a potential therapeutic for neurodegeneration. CIP specifically targets the hyperactivated state of CDK5 as mediated by p25/p29, while allowing normal activation of CDK5 by p35/p39. CDK5 inhibitory peptide (CIP) was found to inhibit the hyperactivation of CDK5 by p25 overexpression *in vivo*, which reduced neurodegeneration and improved cognitive function of transgenic mice, without affecting neurodevelopment [70]. In the future, CIP could possibly be adapted to treat certain cancers caused by aberrant CDK5 activation.
